# Integrated analysis of Solute carrier family-2 members reveals SLC2A4 as an independent favorable prognostic biomarker for breast cancer

**DOI:** 10.1080/19336950.2021.1973788

**Published:** 2021-09-07

**Authors:** Zhenyu Shi, Jiahao Liu, Fei Wang, Yongqiang Li

**Affiliations:** Department of Predictive Medicine,Institute of Biomedical Informatics, Cell Signal Transduction Laboratory, Bioinformatics Center, Henan Provincial Engineering Center for Tumor Molecular Medicine, School of Software, School of Basic Medical Sciences, HenanUniversity,Kaifeng,China

**Keywords:** Breast cancer, *SLC2A4*, prognosis, biomolecular network, DNA methylation

## Abstract

Most of *Solute carrier family-2* (SLC2) members play a key role of facilitative transporters, and glucose transporter (GLUT) proteins encoded by *SLC2s* can transport hexoses or polyols. However, the function and mechanism of *SLC2s* remain unclear in human cancers. Here, we explored the dysregulated expression, prognostic values, epigenetic, genetic alterations, and biomolecular network of *SLC2s* in human cancers. According to the data from public-omicsrepository, *SLC2A4* (*GLUT4*) was found to be significantly downregulated in most cancers, and higher messenger RNA (mRNA) expression of *SLC2A4* significantly associated with better prognosis of breast cancer (BRCA) patients. Moreover, DNA hypermethylation in the promoter of *SLC2A4* may affect the regulation of its mRNA expression, and *SLC2A4* was strongly correlated with pathways, including the translocation of *SLC2A4* to the plasma membrane and PID INSULIN PATHWAY. In conclusion, these results provide insight into *SLC2s* in human cancers and suggest that *SLC2A4* could be an unfavorable prognostic biomarker for the survival of BRCA patients.

## Introduction

Saccharides are essential energy sources and carbon sources for human body, which can participate in the formation of various organizational structures. Rapid proliferation, invasion, and other behaviors determine that cancer has a great demand for saccharides. Following Warburg effect, many cancer cells uptake a large number of glucose and regulate their energy source from mitochondrial oxidative phosphorylation to a glucose-dependentglycolytic pathway for maintaining proliferation. [1,2] Solute carrier family-2 (SLC2) can encode glucose transporter (GLUT) protein, which was the memberof MFS (Major Facilitator Superfamily)superfamily, and it is now clear that 14 SLC2 members are *SLC2A1, SLC2A2, SLC2A3, SLC2A4, SLC2A5, SLC2A6, SLC2A7, SLC2A8, SLC2A9, SLC2A10, SLC2A11, SLC2A12, SLC2A13*, and*SLC2A14*, which separately encodes*GLUT1, GLUT2, GLUT3, GLUT4, GLUT5, GLUT6, GLUT7, GLUT8, GLUT9, GLUT10, GLUT11, GLUT12, HMIT*, and *GLUT14*[3,4]. All GLUTs seemed to transport hexoses or polyols when ectopically expressed[5]. The 14 GLUT proteins are comprised of 500 amino acid residues and can be categorized into three classes based on sequence similarity: Class 1 (*GLUTs 1–4* and *14*); Class 2 (*GLUTs 5*, *7*, *9*, and *11*); and Class 3 *(GLUTs 6*, *8*, *10*, *12*, and *HMIT*) [6]. To date, *GLUT1-5* had been researched most extensively and deeply [5].

Several studies have found aberrant expressions and unique mechanisms in some members of *GLUTs* family. For example, *GLUT1* overexpression promoted the glycolysis process in many human cancers, such as gastric adenocarcinoma and breast cancer carcinoma and adenocarcinoma [7]. Besides, *GLUT1* expression was regulated by long non-codingRNA HOX transcript antisense RNA and microRNA miR-150 [8,9]. *GLUT2* transporters showed the activity in hepatocellular carcinoma cell, and the knockdown of *GLUT2* can induce the apoptosis in HepG2 cells [10]. Moreover, *GLUT3* may play an important role in proliferation and apoptosis in human cancers [11]. *GLUT4* was found to be correlated with 18 F-flurodeoxyglucose uptake in gastrointestinal stromal tumor [12]. Furthermore, *GLUT5* can act as fructose transporter in *vivo* in human breast cancer [13]. However, the prognostic significance and molecular mechanisms of *SLC2s* remain unclear.

Nowadays, with the development of high throughput technologies, as well as public attention to cancer genomes, researchers can use The Cancer Genome Atlas (TCGA) to explore the molecular mechanisms and genomic changes of a variety of human tumors. Thus, cancer prevention, diagnosis, and precision therapy were greatly speeded up. In this study, we analyzed the messenger RNA (mRNA) expression, gene mutation, and methylation modification of *SLC2s* in human cancers based on TCGA datasets and discussed their prognostic value and gene regulatory network in breast cancer.

## Materials and method

### SLC2s-mRNA expression analysis

The oncomine (www.oncomine.org) database was used to analyze the expression of *SLC2s*-mRNA between different cancer tissues [14]. The cut-off of *p*-value and fold change were as follows: *p*-value: 0.01, fold change: 1.5, gene rank: 10%. In addition, UALCAN (http://ualcan.path.uab.edu) is an interactive web resource to perform in-depth analyses of gene expression between tumor and normal samples based on individual clinicopathologic features from the TCGA data. The mRNA expression of *SLC2s* in different cancer subtypes and grades/stages was also analyzed by UALCAN[15].

### Kaplan–Meier survival analysis

The relevance between the *SLC2s*’ expression and prognosis was analyzed by Kaplan–Meier plotter (http://kmplot.com/analysis)[16]. “Overall survival (OS),” “auto-select best cut-off,” and “only JetSet best probe set” was chosen for calculating and draw Kaplan–Meier survival curve. Best cut-off values were calculated by all possible cut-off values between the lower and upper quartiles, and the best generated execution threshold was used as a cut-off (Supplementary Table 1). Statistical significance was determined by log-rank *p*-value, and hazard ratios (HRs) with 95% CIs are displayed. The Affymetrix probeset IDs of SLC2 family members in breast cancer, lung cancer, and stomach cancer are as follows: *SLC2A1*: 201249_at; *SLC2A2*: 206535_at; *SLC2A3*: 202499_s_at; *SLC2A4*: 206603_at; *SLC2A5*: 204430_s_at; *SLC2A6*: 220091_at; *SLC2A8*: 218985_at; *SLC2A9*: 219991_at; *SLC2A10*: 221024_s_at; *SLC2A11*: 232167_at; *SLC2A12*: 244353_s_at; *SLC2A13*: 227176_at; and *SLC2A14*: 216236_s_at. The Affymetrix probeset ID of *SLC2A7* was not found in KM plotter. Furthermore, GSE62254 dataset was excluded when KM plots were generated for *SLC2s* in stomach cancer because of GSE62254 having markedly different characteristics (longer survivals, shifted expression) than the other datasets.

### Mutations and copy-numberalterations analysis

cBioPortal (www.cbioportal.org) is an online open access website resource that can be used to interactively explore multidimensional cancer genomics datasets [17]. Gene mutations and copy number alternation of *SLC2A4* was analyzed from Invasive Breast Cancer (TCGA, firehorse legacy, 1101 patients/1108 samples) in cBioPortal.

### Methylation modification analysis

MEXPRESS (https://mexpress.be) was used to analyze the correlation between DNA methylation and *SLC2A4* mRNA expression in 1268 breast invasive carcinoma samples [18]. Besides, MethHC (http://MethHC.mbc.nctu.edu.tw) was used to study the relationship of DNA methylation in the promoter and *SLC2A4*-mRNA expression for 839 breast invasive carcinoma samples [19].

### Gene regulatory networks analysis

The Search Tool for the Retrieval of Interacting Genes/Proteins (STRING,https://www.string-db.org) was used to construct the interaction network of *SLC2A4* with a confidence score of 0.4 [20]. Additionally, Metascape (https://metascape.org) was chosen for gene ontology and pathway enrichment analysis of the genes related to *SLC2A4*[21].

## Results

### SLC2s-mRNA expression in breast cancer

**Figure 1.**
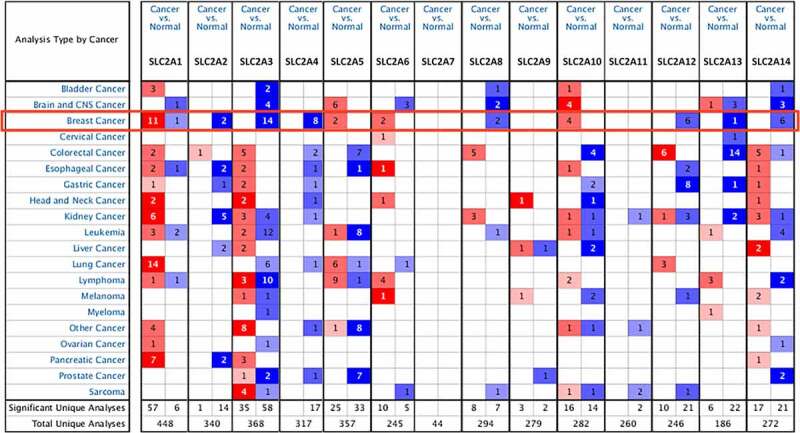
The mRNA levels of SLC2s in different cancer types (ONCOMINE database)

Using the Oncomine database, the mRNA expression of *SLC2s* in 20 cancer types was analyzed (Figure 1). Results revealed that *SLC2A1*, *3*, *4*, *5*, *6*, *10*, *13* and *14* were abnormally expressed in most cancer types. Among them *SLC2A1*, *3*, *4* and *14* has the most data available in breast cancer. Compared with normal tissues, *SLC2A3*, *4* and *14* were significantly downregulated in breast cancer. In the Curtis dataset, *SLC2A3* mRNA expression was observed 2.665-fold decrease in invasive lobular breast carcinoma samples, while *SLC2A4* downregulation was found in invasive breast carcinoma samples with a fold change of 5.226 from TCGA Breast dataset[22]. TCGA Breast datasets also showed 2.617-fold decrease of *SLC2A14* mRNA expression in mucinous breast carcinoma samples. Concurrently, *SLC2A1* was significantly up-regulatedin breast cancer. In Zhao Breast dataset, *SLC2A1* was overexpressed in invasive ductal breast carcinoma compared with normal tissues with a fold change of 2.800[23]. However, in Finak’s datasets, *SLC2A1* was significantly down-regulated in invasive breast carcinoma stroma sample with a 3.780-fold change[24].

The gene rank was analyzed by percentile of target gene in the top of all genes measured in each research. Red and blue color scales determined by the best gene rank percentile indicate gene expression upregulation and downregulation, respectively. Cutoff of*p*-value and fold change defined as:*p*-value: 0.01, fold change: 2, gene rank: 10%, data type: mRNA.

**Figure 2.**
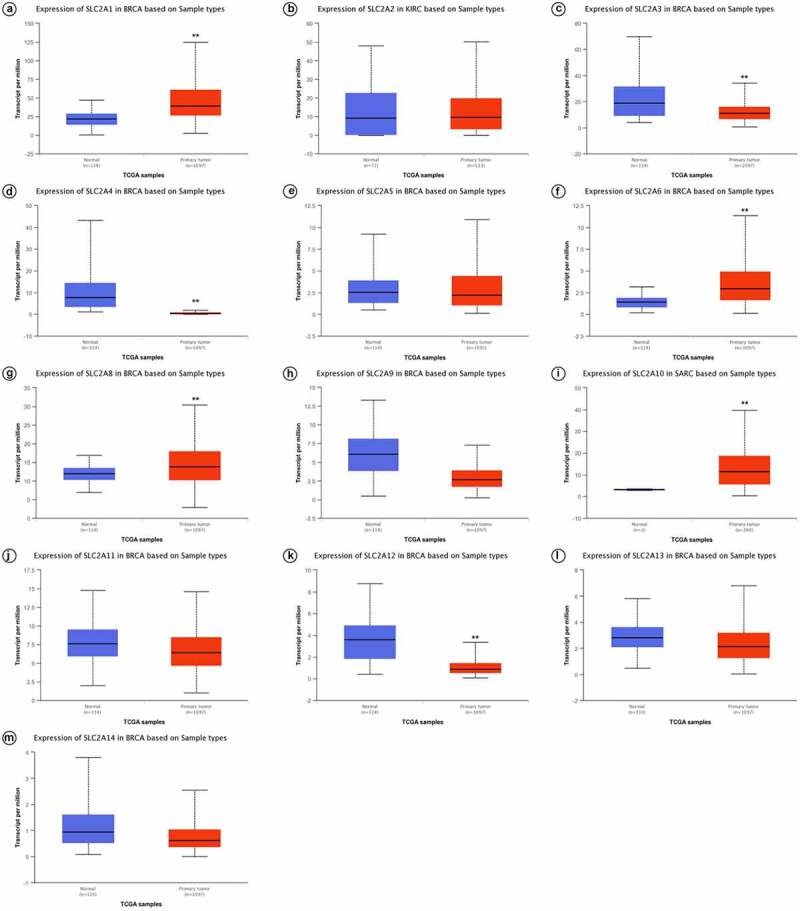
Box-whisker plots showing the mRNA levels of SLC2s in breast invasive carcinoma samples based on major cancer stages from the UALCAN database

UALCAN was further used to investigate the expression of *SLC2s* in breast cancer. As shown in Figure 2 and Table 1, *SLC2A1*, *3*, *4*, *6*, *8*, *9*, *10* and *12* were significantly dysregulated in breast cancer (*p <*0.01). We found that *SLC2A1* (*p <*1.00E-12), *SLC2A6* (*p <*1.00E-12), *SLC2A8* (*p =*1.62E-12) and *SLC2A10* (*p =*1.62E-12) were significantly up-regulated 1.80-fold, 2.10-fold, 1.15-fold and 1.85-fold, respectively. *SLC2A3* (*p =*3.01E-07), *SLC2A4* (*p* =1.04E-11), *SLC2A9* (*p =*4.44E-16) and *SLC2A12* (*p* =1.62E-12) were also found to be significantly downregulated 1.71-fold, 20.91-fold, 2.28-fold and 4.07-fold, respectively.

**Table 1.** Significant changes of SLC2s expression in transcript level compared BRCA tissues with normal tissues. *P*<0.05 was treated as significant

Gene	mRNA expression	*p*-value	Fold change
SLC2A1	High	1E-12	1.80
SLC2A6	High	1E-12	2.10
SLC2A8	High	1.62E-12	1.15
SLC2A10	High	1.62E-12	1.85
SLC2A3	Low	3.01E-07	1.71
**SLC2A4**	**Low**	**1.04E-11**	**20.91**
SLC2A9	Low	4.44E-16	2.28
SLC2A12	Low	1.62E-12	4.07

(a)*SLC2A1*; (b)*SLC2A2*; (c)*SLC2A3*; (d)*SLC2A4*; (e)*SLC2A5*; (F)*SLC2A6*; (g)*SLC2A8*; (h)*SLC2A9*; (i)*SLC2A10*; (j)*SLC2A11*; (k)*SLC2A12*; (l)*SLC2A13*; (m)*SLC2A14*. **p < 0.01

### Prognostic value of SLC2s expression in breast cancer patients

**Figure 3.**
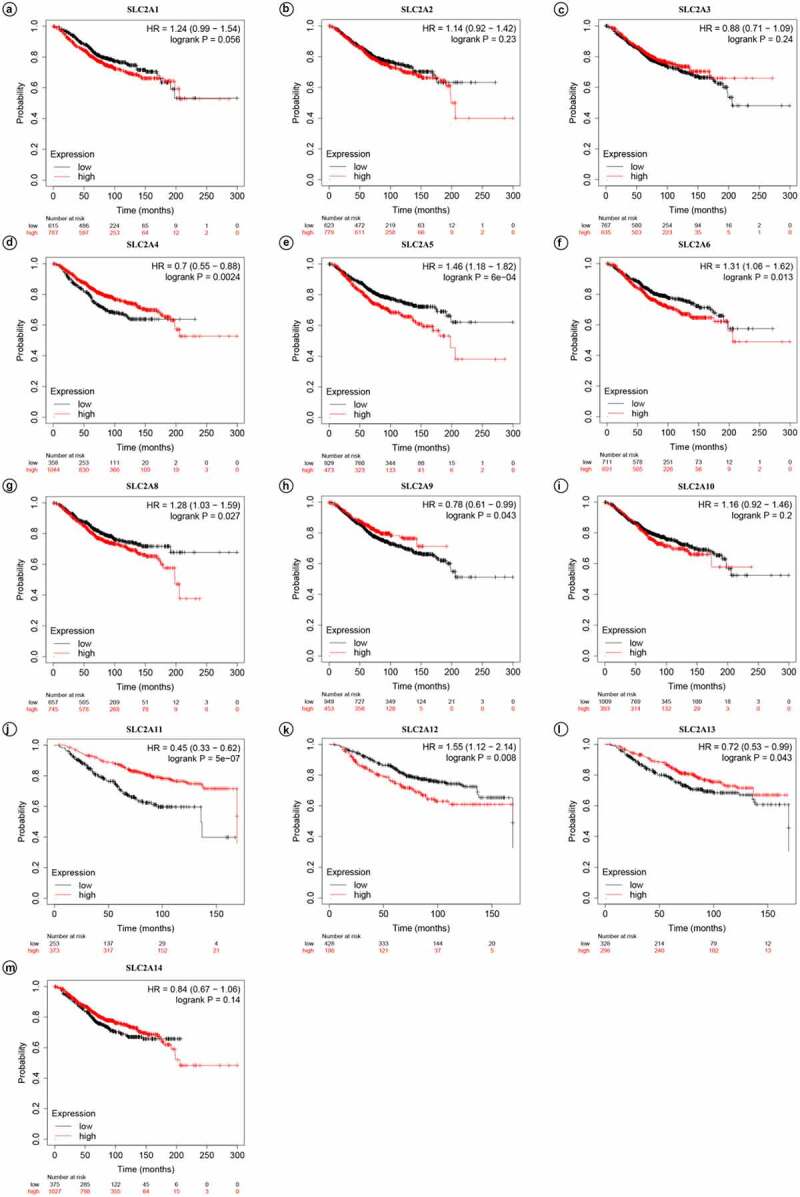
**Prognostic values of SLC2s for OS in all breast cancer patients (**1402 invasive carcinoma patients).*p*-values were calculated by log-ranktest. HRs with 95% confidence intervals (CIs) are displayed. (a)*SLC2A1*; (b)*SLC2A2*; (c)*SLC2A3*; (d)*SLC2A4*; (e)*SLC2A5*; (f)*SLC2A6*; (g)*SLC2A8*; (h)*SLC2A9*; (i)*SLC2A10*; (j)*SLC2A11*; (k)*SLC2A12*; (l)*SLC2A13*; (m)*SLC2A14.*

Next, KM plotter was used to investigate the prognostic values of the mRNA expression of *SLC2s* in breast cancer patients (Figure 3 and Table 2). Results showed that higher mRNA expression of *SLC2A4* and *SLC2A11* significantly associated with better prognosis of breast cancer patients (HR: 0.7 [0.55–0.88];*p* =0.0024 and HR: 0.45 [0.33–0.62];*p* =5E-07, respectively). Moreover, higher mRNA expression of *SLC2A5* and *SLC2A12* significantly associated with worse prognosis of breast cancer patients (HR: 1.46 [1.18–1.82];*p* = 6E-04 and HR: 1.55 [1.12–2.14];*p* = 0.008, respectively). Additionally, there is a certain interaction between abnormal mRNA expression of *SLC2A6, SLC2A8, SLC2A9* and prognosis in breast cancer (HR: 1.13 [1.06–1.62];*p* = 0.013, HR: 1.28 [1.03–1.59];*p* = 0.027 and HR: 0.78 [0.61–0.99];*p* = 0.043, respectively). In summary, the results indicated that *SLC2A4* may be exploited as the most useful biomarkers of *SLC2s* for prediction of breast cancer patients’ survival.

**Table 2.** SLC2 family members with significant prognostic values in breast cancer patients

Gene	Patients number at risk	mRNA expression level	HR	95% CI	Prognostic outcome	*p*-value
**SLC2A4**	**1044**	**High**	**0.7**	**0.55–0.88**	**Better**	**0.0024**
	**358**	**Low**				
SLC2A5	473	High	1.46	1.18–1.82	Worse	6E-04
	929	Low				
SLC2A11	373	High	0.45	0.33–0.62	Better	5E-07
	253	Low				
SLC2A12	198	High	1.55	1.12–2.14	Worse	0.008
	428	Low

**Figure 4.**
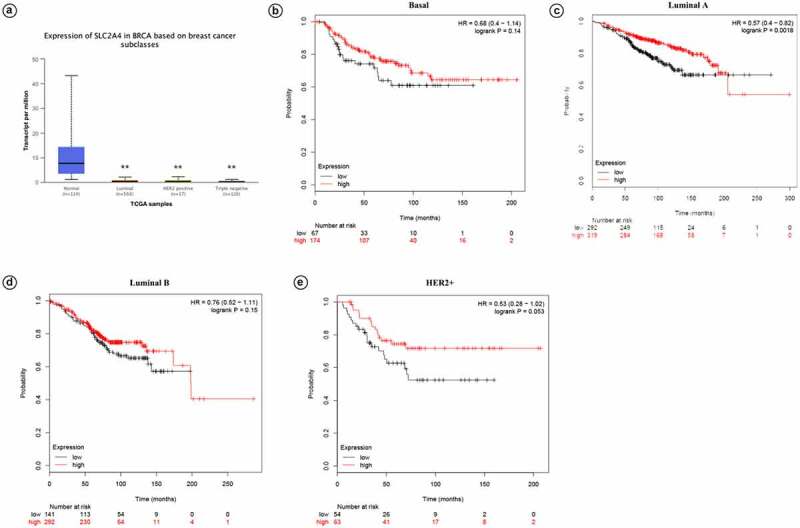
Prognostic values of *SLC2A4* in breast cancer patients with different intrinsic subtypes

Expression of *SLC2A4*-mRNA in different major subclasses of TCGA breast cancer samples are shown as Figure 4. Compared with normal tissues, *SLC2A4* were significantly reduced in luminal, HER2 positive and triple negative breast cancers (*p* = 1.19E-11;*p* = 2.43E-11;*p* = 6.70E-12, respectively). Meanwhile, KM plotter was used to access prognostic values of *SLC2A4* in different intrinsic subtypes. Results showed that *SLC2A4* high expression is associated with better prognosis in 4 intrinsic subtypes (Basal; HR = 0.68 [0.4 − 1.14];*p* = 0.14; Luminal A; HR = 0.57 [0.4 − 0.82];*p* = 0.0018; Luminal B; HR = 0.76 [0.52 − 1.11];*p* = 0.15; HER2+; HR = 0.53 [0.28 − 0.1.02];*p* = 0.053).

(a) mRNA expression of*SLC2A4*in breast cancer patients with different intrinsic subtypes, including luminal, HER2 positive, and triple negative breast cancer patients. Then*SLC2A4*was plotted for different intrinsic subtypes of breast cancer. (b) Basal breast cancer. (c) Luminal A breast cancer. (d) Luminal B breast cancer. (E) HER2+ breast cancer; **p < 0.01.

**Figure 5.**
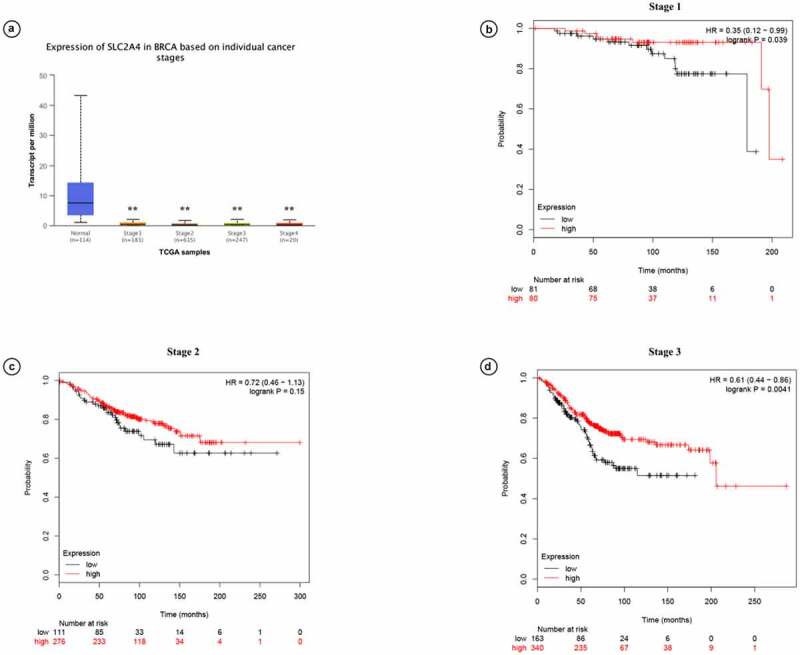
Prognostic values of *SLC2A4* in breast cancer patients with different cancer grades

Prognostic values of *SLC2A4* in breast cancer patients with different cancer stages were also investigated by using UALCAN (Figure 5). We found that mRNA expression of *SLC2A4* was significantly downregulated in all individual cancer stages (*p* < 0.01). KM plotter analysis further demonstrated that high expression of *SLC2A4* is related with better prognosis in grade I and III (HR = 0.35 [0.12 − 0.99];*p* = 0.039; HR = 0.61 [0.44 − 0.86];*p* = 0.0041). However, there was no significant correlation between mRNA expression of *SLC2A4* and prognosis in grade II (HR = 0.72 [0.46 − 0.1.13];*p* = 0.15). Overall, this result showed that *SLC2A4* is a potential prognostic biomarker for breast cancer patients.

(a) mRNA expression of SLC2A4 in breast cancer patients with different cancer stages, including stages 1, 2, and 3. (b) Stage 1. (c) Stage 2. (d) Stage 3. **p < 0.01.

### Relationship between DNA hypermethylation in the promoter of SLC2A4 and SLC2A4-mRNA expression in breast cancer

**Figure 6.**
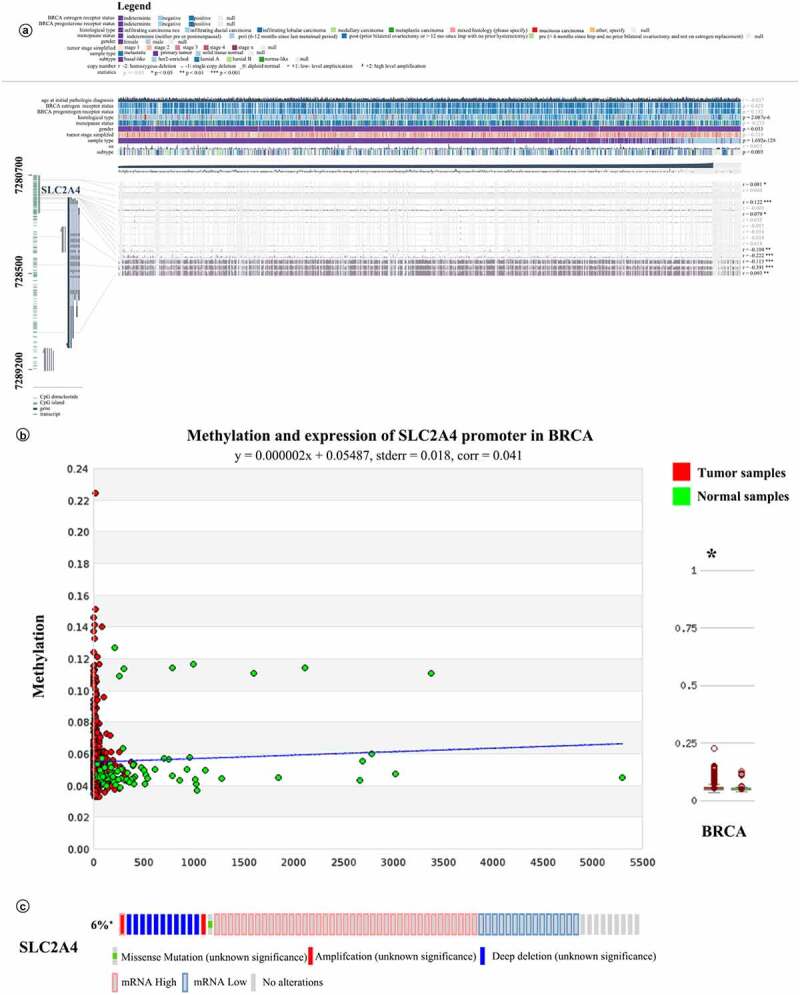
Impact of epigenetic and genetic alterations on *SLC2A4*-mRNA expression in breast cancer

We use MEXPRESS to assess whether DNA hypermethylation in the promoter of *SLC2A4* was related to mRNA expression of *SLC2A4.* As shown in Figure 6, according to the Pearson correlation coefficient, mRNA expression of *SLC2A4* was positively correlated with its DNA methylation in the promoter (probe ID: cg07287120, *r* = 0.122; probe ID: cg03670302*, r* = 0.093; all *p* < 0.01) and can be negatively correlated with its DNA methylation in the promoter (probe ID: cg17663577, *r* = −0.104; probe ID: cg21994579, *r* = −0.222; probe ID: cg06891043, *r* = −0.113; probe ID: cg27067158,*r* = −0.391; all *p* < 0.01). The expression level of *SLC2A4* in breast cancer tissues was lower than that in normal samples. Besides, DNA methylation in *SLC2A4* promoter region was significantly related to histological type, sample type, and subtype (*p* = 2.087E-6,*p* = 1.692E-129 and*p* = 0.003, respectively). We further compared DNA hypermethylation in the promoter of *SLC2A4* in breast cancer patients to normal samples; data from MethHC showed that there were significant differences between the two groups (*p* < 0.05). All in all, these results demonstrated that DNA hypermethylation in the promoter of *SLC2A4* can affect the regulation of its mRNA expression.

(a) Visualization of the TCGA data for*SLC2A4*mRNA expression in breast cancer using MEXPRESS. 1268 breast invasive carcinoma samples were included. The methylation data for each probe was determined using Infnium HumanMethylation 450 microarray. Gene expression data were derived from IlluminaHiSeq RNASeqV2. The samples are ordered by their expression level. The significance of the relation (correlation coefficient or*p*-value) between each row of data (clinical, expression or methylation) was shown in the right side. (b) The promoter methylation levels of*SLC2A4*in 839 breast invasive carcinoma samples were analyzed by MethHC database. (c) OncoPrint of*SLC2A4*alterations in breast cancer. Tumor samples are shown in columns.

To explore the relationship between low mRNA expression of *SLC2A4* and genetic alterations, we used cBioPortal to analyze the mutations, copy-number alterations, and mRNA expression transformations of *SLC2A4*. As shown in Figures 6(c), 6% (68) of 1108 breast invasive carcinoma samples have alternations (*p* < 0.05). Among them, 1 sample was missense mutation (unknown significance), 1 sample was amplification (unknown significance), 11 samples were deep deletion (unknown significance), 15 samples were mRNA low, and 39 samples were mRNA high. Interestingly, 1 sample was both amplification and mRNA high. These outcomes indicated that alternations of *SLC2A4* were most mutually independent.

### Biomolecular network regulated by SLC2A4

**Figure 7.**
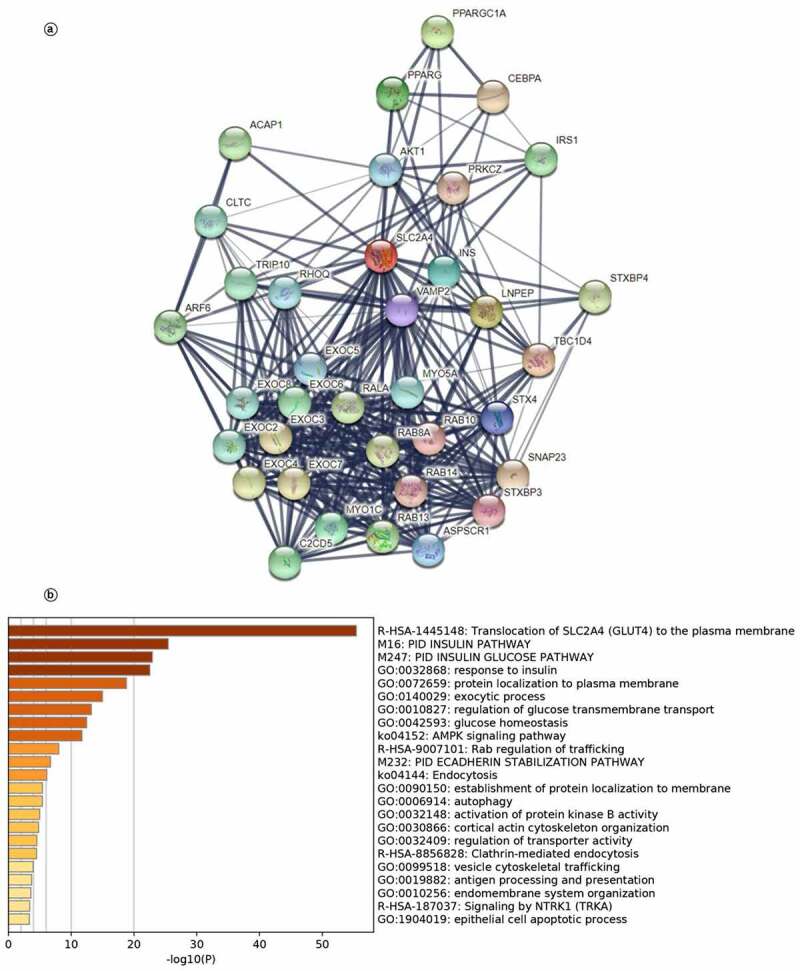
*SLC2A4*-regulated biomolecular network

We further searched potential regulated genes to seek the regulation function of *SLC2A4* in tumorigenesis and tumor progression. As shown in Figure 7 and Supplementary Table 2, we found 35 genes were closely related to *SLC2A4* from functional interaction network analysis using STRING, including *PPARGC1A, PPARG, CEBPA, ACAP1, AKT1, PRKCZ, IRS1, CLTC*, and *ARF6*,

(a) Protein–protein interaction networks. Line thickness indicates the strength of data support. (b) The 35*SLC2A4-*relatedgenes were analyzed by Metascape, and 20 clusters were found (*p* < 0.01).

*TRIP10, RHOQ, VAMP2, INS, LNPEP, TBC1D4, STXBP4, EXOC2, EXOC3, EXOC4, EXOC5, EXOC6, EXOC7, EXOC8, C2CD5, MYO1C, RAB13, ASPSCR1, STXBP3, SNAP23, STX4, MYO5A, RALA, RAB8A, RAB14,* and *RAB10*. Next, gene ontology and pathway enrichment analysis for these 35 genes were performed in Metascape. As shown in Figures 7(b), 20 gene sets were significantly enriched. The most significantly enriched five gene sets were translocation of *SLC2A4* (*GLUT4*) to the plasma membrane and protein localization to plasma membrane. These results demonstrated that *SLC2A4* was related with cellular material transportation and insulin in breast cancer. In addition, *SLC2A4* may play an important role in breast cancer, including AMPK pathway, glucose homeostasis, and protein localization.

## Discussion

In this study, we systematically explore the mRNA expression, prognostic value, epigenetic and genetic alterations, and gene regulatory network of *SLC2A4* in human cancers. Results from our study showed that the mRNA expression of *SLC2A4* was significantly decreased in BRCA tissues, and high level of *SLC2A4*-mRNA was significantly relevant to better prognosis in breast cancer patients. Compared with normal tissues, *SLC2A4* was significantly downregulated in patients with all subtypes and stages of BRCA, and high level of *SCL2A4* in luminal A breast cancer and stage III breast cancer can significantly predict better prognosis. Additionally, *SLC2A4* can also abnormally express in other types of cancers. According to the latest cancer epidemic trend from China National Cancer Center, we also investigated the relevance between the expression and prognosis of *SLC2A4* in lung cancer, liver cancer, and stomach cancer [25]. Results are shown in Supplementary Table 3. It suggested that *SLC2A4* can be a common prognostic biomarker in specific stages of multiple cancers. The latest progress in tumor epigenetics shows that DNA methylation, histone modification, and non-codingRNA play an important role in regulating gene expression and chromatin structure, which may lead to the occurrence and development of cancer [26]. In addition, the increase of DNA copy number will also lead to the increase of gene mRNA expression. Above results indicated that *SLC2A4* may be an important biomarker to promote the development of accurate diagnosis and prognosis.

As one of the sugar transporter proteins in human genome, *GLUT4* regulates glucose transport and is encoded by *SLC2A4*[27,28].*GLUT4* plays an essential role in maintaining body glucose homeostasis and is regulated by insulin. *GLUT4* expression was downregulated in adipocytes in obesity and was upregulated in adipocytes and muscle cells in response to exercise [29]. *GLUT4* was expressed differently among normal humans and those with obesity and diabetes, and the overexpression of *GLUT4* in skeletal muscle can alter substrate utilization and improve the benefits of insulin [30,31]. Besides, three studies have shown that *GLUT4* may play a potential role in tumorigenesis and progression [32–34]. *GLUT4* downregulation may inhibit glucose uptake and induce metabolic reprogramming; research also found that GLUT4 downregulation can suppress cell proliferation and critically decrease cell viability under hypoxic conditions, especially in MCF7 and MDA-MB-231 breast cancer cells [33]. Besides, 7 antimicrobial peptides (RAB1-7) became anti-cancerdrugs by inhibiting *SLC2A4* to impair the energy gained by cancer cells during angiogenesis [32]. Another study demonstrated that high glucose increased *SLC2A4* and *VEGF*/*VEGFR* expression by upregulating estrogen receptor and further promoted epithelial–mesenchymal transition process and accelerated the development of uterus endometrial cancer [34]. Moreover, by targeting *GLUT4*, the silence of krüppel-like transcription factor 8 (*KLF8*) expression decreased the glycolysis rate of gastric cancer cells in *vitro*[35]. However, the physiological and pathophysiological mechanisms of *GLUT4* protein are still unclear. Multiple studies revealed that *GLUT4* was associated with the regulation adipogenesis and blood glucose regulation [36–40]. Interestingly, exercise may be a key role of affecting the translocation and expression of *GLUT4*; exercise can activate AMPK, PPAR β positive feedback loop and PGC-1 α, and further upregulate *GLUT4* expression to enhance glucose uptake by tissue cells [41–43]. Some extracts from plants, including quercetin and *Moringa concanensis* nimmo extracts, can upregulate the expression of *GLUT4*[44,45].

We performed functional enrichment analysis of 35 *SLC2A4*-related genes and discovered some important pathways that may play a key role in tumorigenesis and progression. Pathways that *SLC2A4*-related genes most significantly enrichedinclude translocation of *SLC2A4* (*GLUT4*) to the plasma membrane, PID INSULIN PATHWAY, PID INSULIN GLUCOSE PATHWAY, response to insulin and protein localization to plasma membrane. These pathways may contribute to the discovery of novel mechanisms of *SLC2A4*.

Insulin is a kind of protein hormone secreted by islet β cells, and it is also the only hormone that can reduce blood glucose in human body. Increasing evidence suggested that the upregulated insulin was associated with tumorigenesis and cancer growth [46–48]. Although increased insulin production was a common phenomenon during cancer development, the insulin resistance also occurred in the normal tissues and lead to alterations in carbohydrate and lipid metabolism [48]. Insulin and insulin-like growth factor receptors may play a pivotal role in cell fate determination, and they can regulate cell proliferation, differentiation, apoptosis, glucose transport, and energy metabolism [47]. When exposed to hyperinsulinemia, cancer cells gain a selective growth advantage compared to normal tissues [46]. It has been found that the higher breast cancer incidence and higher all-cause mortality after breast cancer were significantly correlated with higher levels of insulin resistance in postmenopausal women [49,50]. Analyzing protein–protein interaction network in STRING database, *SLC2A4* also participates in the translocation of *SLC2A4* (*GLUT4*) to the plasma membrane and protein localization to plasma membrane through enriched proteins such as *EXOC5* and *C2CD5*, which play an important role in human cancers. However, most of them were rarely studied in the pathways we found. Another important pathway related to *SLC2A4* was AMP-activated proteinkinase (AMPK) pathway, which was a highly conserved and widely expressed energy balance regulator in eukaryotic cells and play a key role in carcinogenesis and cancer drug resistance [51–53]. Seventeen related genes we found were closely associated with AMPK signaling pathway, including *AKT1, RAB8A,* and *IRS1*. Studies demonstrated that Insulin receptor substrate 1 (*IRS1*) promoted tumor growth in colorectal cancer targeted by miR-30a-5p and was stabilized by RNA-bindingprotein lin-28 homolog B (*LIN28B*) [54]. It is important to correlate the data with the insulin levels of each cancer patient. If the cancer patient had Type 2 diabetes characterized by insulin resistance of course *GLUT4* levels will be reduced. In that case, *SLC2A4* expression level has no prognostic level for cancer outcome but diabetes and increased insulin secretion have. Reduced *GLUT4* expression is only a byproduct of high chronic plasma insulin levels.

The most common genes enriched in 23 pathways were *ARF6, AKT1, VAMP2, MYO1C, MYO5A,* and *RAB8A*, which were found in 14, 13, 13, 12, 12, and 12 gene sets, respectively. Other genes such as *INS, RNB10, PRKCZ, RAB13,* and *RAB10* can also participate in most pathways. Li et al. [55] indicated that ARF6 can regulate the functions of membrane traffic, and overexpression of *ARF6* was correlated with poor prognosis in multiple invasive cancers, such as triple-negative breast cancer and invasive clear cell renal cell carcinoma. Riggio et al. [56] demonstrated that *AKT1* promoted cell proliferation via upregulating cyclin-dependent kinase 1 and S6, and C-X-C motif chemokine receptor 2 (*CXCR2*) promoted breast cancer metastasis and chemoresistance by inhibiting *AKT1* and activating COX2 (Cyclooxygenase 2). Moreover, Wang et al. [57] showed that the expression of vesicle-associated membrane protein 2 (*VAMP2*) was negatively regulated by miR-493-5p and further suppressed the proliferation and migration in liver cancer. Several studies had showed that Myosin 1 C (*MYO1C*) may play a key role in regulatingautophagosome–lysosome fusion through F-actin remodeling, and miR-137 overexpression inhibited the cell migration, proliferation by targeting Krűppel-likefactor 12 (*KLF12*) and *MYO1C* in gastric cancer cell lines [58,59]. Besides, myosin VA (*MYO5A*) may play a crucial role of diagnosis and prognosis in glioblastoma multiforme and gastric cancer [60,61]. Li et al. [62] found that Rab8a can regulate *GLUT4* trafficking in muscle and adipose cells, and the suppression of Rab8A inhibited insulin-stimulated *GLUT4* translocation. These results revealed that *SCL2A4* had undeniable potential functions in the development and progression of human cancers.

There were some limitations in our research. First, all of our analysis were conducted based on publicly available datasets and further experimental studies consisting of larger sample sizes may contribute to validate our results. Meanwhile, performing abundant investigations will validate whether the bioinformatics results from public datasets is uniform with immunohistochemistry staining or western blot. Second, the study required more comprehensive and detailed analysis; researching all the gene sets enriched by Metascape will help to explore more potential functions and mechanisms of *SLC2A4* in breast cancer. The potential diagnostic and therapeutic role of *SLC2A4* can be assessed in great detail. Finally, we only investigated the most likely prognostic biomarker of *SLC2s* in breast cancer. Biomolecular network of other SLC2 family members will help us to deeply understand the role of *SLC2s* in breast cancer.

## Conclusion

In conclusion, we systematically analyzed the potential function and molecular mechanism of *SLC2s* in human cancer. High expression of *SLC2A4* was significantly correlated with better prognosis in breast cancer patients. Furthermore, the expression of *SLC2A4* mRNA was found to be regulated by DNA hypermethylation. To our knowledge, this is the first study that revealed that *SLC2A4* could be a prognostic biomarker for survivals of breast cancer patients.

## Supplementary Material

Supplemental Material
