# Supplementary material for: Integrated analysis of Solute carrier family-2 members reveals SLC2A4 as an independent favorable prognostic biomarker for breast cancer
Source: Channels (Austin). 2021 Sep 7;15(1):555–68. doi: 10.1080/19336950.2021.1973788 (PMC8425726; doi:10.1080/19336950.2021.1973788)
Supplement: Supplemental Material [file KCHL_A_1973788_SM4009.docx]

**Supplementary Table 1.** Cutoff values used in analysis for SLC2s KM plotter.

| Items | Expression range of the probe | Cutoff value used in analysis | False discovery rate | Patients |
| --- | --- | --- | --- | --- |
| Cutoff values for MFAPs KM plotter in all breast cancer patients | | | | |
| Gene name |  |  |  |  |
| *SLC2A1* | 2 - 1433 | 65 | 100% | 1402 |
| *SLC2A2* | 0 - 163 | 4 | 100% | 1402 |
| *SLC2A3* | 2 - 5574 | 473 | 100% | 1402 |
| *SLC2A4* | 1 - 348 | 11 | 50% | 1402 |
| *SLC2A5* | 6 - 2601 | 185 | 20% | 1402 |
| *SLC2A6* | 3 - 1618 | 72 | over 50% | 1402 |
| *SLC2A7* | - | - | - | - |
| *SLC2A8* | 9 - 892 | 148 | over 50% | 1402 |
| *SLC2A9* | 22 - 4538 | 422 | over 50% | 1402 |
| *SLC2A10* | 34 - 10543 | 2309 | 100% | 1402 |
| *SLC2A11* | 10 - 720 | 173 | 1% | 1402 |
| *SLC2A12* | 3 - 1411 | 102 | over 50% | 1402 |
| *SLC2A13* | 47 - 1792 | 341 | over 50% | 1402 |
| *SLC2A14* | 14 - 2110 | 177 | 100% | 1402 |
|  | | | | |
| Cutoff values for SLC2A4 KM plotter in breast cancer patients with different subtypes | | | | |
| Breast cancer subtypes |  |  |  |  |
| basal | 1 - 225 | 6 | 100% | 241 |
| luminal A | 1 - 270 | 21 | 50% | 611 |
| luminal B | 1 - 348 | 19 | 100% | 433 |
| HER2+ | 1 - 170 | 15 | 100% | 117 |
|  | | | | |
| Cutoff values for MFAP4 KM plotter in breast cancer patients with different stages | | | | |
| Breast cancer grades |  |  |  |  |
| 1 | 1 - 235 | 37 | over 50% | 161 |
| 2 | 1 - 348 | 19 | 100% | 387 |
| 3 | 1 - 306 | 15 | over 50% | 503 |
|  | | | | |
| Cutoff values for MFAP4 KM plotter in lung, liver and stomach cancer | | | | |
| Cancer names |  |  |  |  |
| lung cancer | 1 - 329 | 29 | 1% | 1925 |
| liver cancer | 1 - 339 | 23 | over 50% | 364 |
| stomach cancer | 0 - 2543 | 293 | 20% | 592 |

**Supplementary Table 2.** 35 *SLC2A4* related genes were analyzed by Metascape, and 20 clusters were found to be significantly associated with SLC2A4 (*p* < 0.01).

| GO | Category | Description | Count | % | Log10(P) | Log(q-value) | Gene symbol |
| --- | --- | --- | --- | --- | --- | --- | --- |
| R-HSA-1445148 | Reactome Gene Sets | Translocation of *SLC2A4* (*GLUT4*) to the plasma membrane | 28 | 33.33% | -55.4197 | -51.073 | *AKT1, LNPEP, RAB8A, MYO1C, MYO5A, RAB13, RALA, STX4, STXBP3, VAMP2, SNAP23, C2CD5, TBC1D4, EXOC5, RAB10, EXOC3, EXOC7, RHOQ, RAB14, EXOC6, EXOC2, EXOC4, ASPSCR1, EXOC8, ARF6, CLTC, INS, TRIP10* |
| M16 | Canonical Pathways | PID INSULIN PATHWAY | 26 | 27.27% | -25.4485 | -21.704 | *AKT1, INS, IRS1, PRKCZ, TRIP10, EXOC5, EXOC3, EXOC7, RHOQ, EXOC6, EXOC2, EXOC4, MYO5A, VAMP2, EXOC8, ARF6, CLTC, RALA, ACAP1, RAB8A, STX4, SNAP23, RAB10, RAB14, RAB13, PPARGC1A* |
| M247 | Canonical Pathways | PID INSULIN GLUCOSE PATHWAY | 17 | 38.46% | -22.9268 | -19.279 | *AKT1, INS, LNPEP, PRKCZ, STX4, VAMP2, TRIP10, TBC1D4, RHOQ, STXBP4, RAB8A, RAB13, SNAP23, C2CD5, RAB14, EXOC8, ARF6, IRS1, MYO1C, MYO5A, STXBP3, EXOC4, CLTC, CEBPA, RAB10* |
| GO:0032868 | GO Biological Processes | response to insulin | 17 | 5.61% | -22.5264 | -19.025 | *AKT1, INS, IRS1, RAB8A, MYO1C, MYO5A, PPARG, PRKCZ, RAB13, STXBP3, VAMP2, C2CD5, TBC1D4, RAB10, RHOQ, STXBP4, PPARGC1A* |
| GO:0072659 | GO Biological Processes | protein localization to plasma membrane | 19 | 4.95% | -18.7911 | -15.591 | *AKT1, ARF6, CLTC, INS, RAB8A, MYO5A, PRKCZ, RAB13, STX4, VAMP2, C2CD5, EXOC5, RAB10, RHOQ, MYO1C, EXOC4, IRS1, RALA, EXOC2* |
| GO:0140029 | GO Biological Processes | exocytic process | 20 | 10.47% | -14.9798 | -12.049 | *RAB8A, RAB13, STX4, STXBP3, VAMP2, SNAP23, RAB10, EXOC6, EXOC4, ARF6, PPARG, RALA, C2CD5, TBC1D4, MYO1C, MYO5A, PRKCZ, INS, IRS1, STXBP4* |
| GO:0010827 | GO Biological Processes | regulation of glucose transmembrane transport | 23 | 10.13% | -13.2089 | -10.310 | *AKT1, INS, IRS1, STXBP3, C2CD5, RHOQ, ASPSCR1, STXBP4, PRKCZ, TRIP10, PPARGC1A, EXOC7, MYO5A, VAMP2, TBC1D4, MYO1C, CLTC, PPARG, RAB14, CEBPA, ARF6, LNPEP, STX4* |
| GO:0042593 | GO Biological Processes | glucose homeostasis | 15 | 4.05% | -12.4432 | -9.574 | *AKT1, CEBPA, INS, IRS1, PPARG, STX4, STXBP3, PPARGC1A, ASPSCR1, STXBP4, MYO5A, VAMP2, SNAP23, MYO1C, ARF6* |
| ko04152 | KEGG Pathway | AMPK signaling pathway | 17 | 6.67% | -11.7162 | -8.969 | *AKT1, INS, IRS1, RAB8A, PPARG, RAB10, PPARGC1A, RAB14, CEBPA, ARF6, RAB13, ASPSCR1, CLTC, PRKCZ, VAMP2, MYO5A, RALA* |
| R-HSA-9007101 | Reactome Gene Sets | Rab regulation of trafficking | 8 | 4.84% | -8.01016 | -5.550 | *AKT1, ARF6, RAB8A, RAB13, RAB10, RAB14, CLTC, MYO1C* |
| M232 | Canonical Pathways | PID ECADHERIN STABILIZATION PATHWAY | 4 | 9.76% | -6.69772 | -4.325 | *ARF6, STX4, EXOC3, EXOC4* |
| ko04144 | KEGG Pathway | Endocytosis | 9 | 2.31% | -6.10536 | -3.784 | *ARF6, CLTC, RAB8A, PRKCZ, ACAP1, RAB10, RALA, RAB13, EXOC3* |
| GO:0090150 | GO Biological Processes | establishment of protein localization to membrane | 7 | 1.74% | -5.40218 | -3.155 | *RAB8A, MYO1C, VAMP2, C2CD5, RAB10, EXOC4, STXBP4* |
| GO:0006914 | GO Biological Processes | autophagy | 7 | 1.30% | -5.40062 | -3.155 | *AKT1, CLTC, RAB8A, PPARGC1A, EXOC7, EXOC4, EXOC8* |
| GO:0032148 | GO Biological Processes | activation of protein kinase B activity | 17 | 8.82% | -4.98164 | -2.788 | *AKT1, INS, PRKCZ, PPARG, PPARGC1A, ARF6, RALA, MYO1C, RAB14, IRS1, STX4, VAMP2, STXBP3, SNAP23, CEBPA, TBC1D4, ACAP1* |
| GO:0030866 | GO Biological Processes | cortical actin cytoskeleton organization | 10 | 7.69% | -4.79959 | -2.635 | *ARF6, RAB13, RHOQ, MYO1C, MYO5A, RALA, TRIP10, CLTC, RAB8A, EXOC5* |
| GO:0032409 | GO Biological Processes | regulation of transporter activity | 9 | 1.72% | -4.53038 | -2.407 | *INS, MYO5A, PPARG, VAMP2, PPARGC1A, CEBPA, CLTC, AKT1, RHOQ* |
| R-HSA-8856828 | Reactome Gene Sets | Clathrin-mediated endocytosis | 4 | 2.74% | -4.48438 | -2.369 | *ARF6, CLTC, VAMP2, TRIP10* |
| GO:0099518 | GO Biological Processes | vesicle cytoskeletal trafficking | 3 | 4.00% | -3.94442 | -1.933 | *MYO1C, MYO5A, PRKCZ* |
| GO:0019882 | GO Biological Processes | antigen processing and presentation | 6 | 1.72% | -3.7062 | -1.727 | *CLTC, LNPEP, SNAP23, RAB10, STX4, AKT1* |

**Supplementary Table 3. Prognostic values of *SLC2A4* in lung cancer, liver cancer and stomach cancer patients with stage Ⅰ, Ⅱ, Ⅲ and Ⅳ.**

| Cancer type | Patients number  at risk | *SLC2A4* expression  level | Stage | HR | 95% CI | Prognostic  outcome | p-value |
| --- | --- | --- | --- | --- | --- | --- | --- |
| Lung cancer | 218 | High | 1 | 1.97 | 1.49-2.6 | Worse | 1.10E-06 |
|  | 359 | Low |  |  |  |  |  |
|  | 69 | High | 2 | 1.83 | 1.23-2.72 | Worse | 0.0026 |
|  | 175 | Low |  |  |  |  |  |
|  | 36 | High | 3 | 1.44 | 0.82-2.54 | Worse | 0.2 |
|  | 34 | Low |  |  |  |  |  |
|  | 4 | - | 4 | - | - | - | - |
| Liver cancer | 47 | High | 1 | 0.49 | 0.22-1.09 | Better | 0.076 |
|  | 123 | Low |  |  |  |  |  |
|  | 24 | High | 2 | 0.13 | 0.03-0.57 | Better | 0.0017 |
|  | 59 | Low |  |  |  |  |  |
|  | 59 | High | 3 | 0.35 | 0.18-0.656 | Better | 0.00065 |
|  | 24 | Low |  |  |  |  |  |
|  | 5 | - | 4 | - | - | - | - |
| Stomach cancer | 24 | High | 1 | 2.25 | 0.61-8.32 | Worse | 0.21 |
|  | 15 | Low |  |  |  |  |  |
|  | 16 | High | 2 | 2.03 | 0.86-4.8 | Worse | 0.1 |
|  | 33 | Low |  |  |  |  |  |
|  | 56 | High | 3 | 1.34 | 0.95-1.91 | Worse | 0.096 |
|  | 161 | Low |  |  |  |  |  |
|  | 25 | High | 4 | 0.49 | 0.26-0.9 | Better | 0.019 |
|  | 49 | Low |  |  |  |  |  |
